# Editorial: Qualitative and quantitative risk assessment of hazardous substances in the workplace

**DOI:** 10.3389/fpubh.2023.1207487

**Published:** 2023-05-31

**Authors:** Meibian Zhang, Gaku Ichihara, Zhijun Zhou, Jianlin Lou, Dongming Wang

**Affiliations:** ^1^National Institute of Occupational Health and Poison Control, Chinese Center for Disease Control and Prevention, Beijing, China; ^2^Department of Occupational and Environmental Health, Faculty of Pharmaceutical Sciences, Tokyo University of Science, Noda, Japan; ^3^School of Public Health, Fudan University, Shanghai, China; ^4^School of Medicine, Huzhou University, Huzhou, Zhejiang, China; ^5^Department of Occupational and Environmental Health, School of Public Health, Tongji Medical College, Huazhong University of Science and Technology, Wuhan, Hubei, China

**Keywords:** risk assessment, hazardous substances, preventive measures, occupational health, risk

Risk refers to the possibility that an event will result in a specific outcome (an unfortunate event or adverse outcome). The definition of risk includes two meanings: the uncertainty of risk and the severity of consequences or the loss caused by events, which can be measured by relevant metrics of possibility and outcome of damage, respectively. The “occupational health risk” can be defined as the possibility of work-related diseases or occupational diseases caused by exposure to occupational hazard factors during occupational activities.

Risk assessment is divided into four classic stages: hazard identification, dose-response relationship assessment, exposure assessment, and risk characterization (1). Occupational health risk assessment (OHRA) is to comprehensively and systematically identify and analyze occupational hazards in the workplace, apply specific risk assessment methods, assess the possibility of work-related diseases or occupational diseases caused by exposure to occupational hazards during occupational activities, predict the level of occupational health risks, and provide a basis for taking appropriate risk control measures (2). Therefore, OHRA is an effective method to control occupational hazardous substances in occupational health protection and is an important content in the occupational health field (3). Many countries have developed their own OHRA criteria or guidelines; however, there is still a distance in establishing an optimal OHRA system. Each risk assessment model has advantages and limitations due to its different technical principles (4). There are many studies on methodologies and practical applications of risk assessment for harmful substances. Several studies have been conducted to examine the strengths and weaknesses of different models and assisted in their further refinement and utility (5).

This Research Topic, “*Qualitative and quantitative risk assessment of hazardous substances in the workplace*” in the “Occupational Health and Safety” section of Frontiers journal, aims to bring together the latest quality articles from researchers working in the field of Occupational Health and Safety and focuses on but not limited to (a) Research advance and policy-making on occupational health risk assessment in the workplace; (b) Development of new risk assessment methods or models for harmful substances; (c) Application of multiple qualitative and quantitative risk assessment methods in critical industries; (d) Comparative studies between different qualitative and quantitative risk assessment methods; (e) Preventive measures and occupational risk management based on risk assessment results.

Under this topic, 13 articles have been successfully published with relevant findings contributing to theoretical research and practice in OHRA. The occupational exposure limit (OEL) is often used as a judgment value for over-risk in the risk assessment. As early as the late 19th century, the concept of OEL was first established in Germany. However, due to the small number of harmful substances with OELs and the need for professional technical institutions to provide occupational health services (e.g., sampling, testing, and evaluation) for enterprises, the technology and cost are high, which cannot meet the management requirements of many small and medium-sized enterprises (SMEs) and evaluation criteria of rapidly increasing chemicals. Therefore, some occupational health risk assessment methods (mainly qualitative) have been developed to predict the risks of chemicals for which there are no OELs. These methods can be practical tools for SMEs to manage their occupational health risks.

In this Research Topic, considering the OEL plays an essential role in the exposure assessment of the risk assessment procedure, Maurer et al. developed an interdisciplinary framework for deriving the OEL based on risk assessment frameworks, including problem formulation, literature review, the weight of evidence considerations, point of departure selection/derivation, application of assessment factors, and derivation of the OEL. Xu et al.  developed a strategy for comparing different OHRA methods in the workplace, considering that different risk levels would be obtained for the same hazardous factor when using different OHRA methods. The evaluation strategy included using the risk ratio (RR) to compare risk levels among six OHRA methods [e.g., the Environmental Protection Agency (EPA), Australian, Romanian, Singaporean, International Council on Mining and Metals (ICMM), and the Control of Substances Hazardous to Health models (COSHH)], analyzing correlations of the RRs of the six OHRA methods, verifying the accuracy of each OHRA method using the inherent risk (IR) of the industry. Huang et al. reported a comprehensive risk assessment model (a grading model) could effectively reflect the total risk level of critical hazards in the electronics industry. They concluded that the grading model has strong practicability. Zhou L. et al. introduced the OHRA methods developed in China using the scoping review. A wide range of OHRA methods was developed in China, including applied, comparative, and optimization studies, and each OHRA method had its strengths and limitations. Their applicability needs to be further tested through more applications in different industries, and comparative studies, optimization studies, and modeling studies are also required.

Moreover, more authors focused on assessing the risk levels of occupational hazards in critical industries or workplaces. Zhu et al. investigated the occupational health risks of n-hexane in electronics industries using multiple OHRA models. They found two semi-quantitative OHRA models developed in China might have stronger practicability for the electronics industry, and they recommended specific control measures for reducing the high health risk of workers (especially for cleaning workers). Shi et al. explored the health risk of benzene-exposed workers in the printing industry applying multiple OHRA methods. They found that the printing and pasting workers suffered a higher risk of benzene exposure and provided preventative measures for controlling the risk. Duan et al. reported the severe hazard risk of silica-dust and industrial noise in the ferrous metal foundry using a risk assessment model developed by the ICMM. In addition, some authors focused on the importance of exposure assessment in risk assessment. Wang et al. reported 31.9% of the individual noise levels exceeded 85 dB(A) of noise OEL, and 53.7% of non-coal mining enterprises were not equipped with HPD for workers, especially in small and micro enterprises, and concluded that noise exposure data was crucial for developing more feasible noise controls. Acramel et al. reported that reporting environmental contamination results to healthcare workers could play an essential role in reducing the occupational exposure to antineoplastic drugs in hospitals. Zhou Z. et al. reported that exposure characteristics of kitchen ultrafine particles were related to kitchen operations and recommended relevant protective measures since the kitchen particles were of high exposure and risk levels. Lari et al. established an exposure assessment procedure for assessing dermal exposure to pesticides among farmers using a dosimeter and hand washing methods and highlighted the importance of protective measures.

Moreover, the other two authors focused on the effectiveness of control measures based on the OHRA result. Wu et al. reported that an engineering renovation could significantly reduce the risk level of Hg in the thermometer industry. Dong et al. reported that improving protective measures in factories with acetylene hydrochlorination and ethylene oxychlorination techniques could significantly reduce risk levels and improve workers' liver health.

Progress of OHRA has been achieved. Future research in OHRA should include: (a) Speed up the formulation of OHRA guidelines. The established system needs to clarify the connotation and extension of OHRA since many occupational health practices (e.g., occupational health technique service for enterprises, physical examination for workers, occupational disease surveillance, and workplace hazardous monitoring programs) may be associated with OHRA. (b) Highlight the OHRA methodology study in the applicability of key industries, comparisons between OHRA methods, and methodology optimization since each method has strengths and weaknesses. A national-level of OHRA database in various industries is needed. Theoretical frameworks for comparative studies between different OHRA models must be improved for analyzing the accuracy, parallel, and correlation among different methods. (c) Strengthen the OHRA popularization and application. The concept and developed risk assessment methodology must be applied to occupational health practices, supervision, and law enforcement based on a new exploration of classification and hierarchical management for enterprises.

## Author contributions

All authors listed have made a substantial, direct, and intellectual contribution to the work and approved it for publication.
